# Plasma Protein Binding Rate and Pharmacokinetics of Lekethromycin in Rats

**DOI:** 10.3390/antibiotics11091241

**Published:** 2022-09-13

**Authors:** Pan Sun, Hongzhi Xiao, Jicheng Qiu, Yuying Cao, Jingyuan Kong, Suxia Zhang, Xingyuan Cao

**Affiliations:** 1 Department of Veterinary Pharmacology and Toxicology, College of Veterinary Medicine, China Agricultural University, Beijing 100193, China; 2 Laboratory of Quality & Safety Risk Assessment for Animal Products on Chemical Hazards (Beijing), Ministry of Agriculture, Beijing 100193, China; 3 Key Laboratory of Detection for Veterinary Drug Residue and Illegal Additives, Ministry of Agricultural, Beijing 100193, China

**Keywords:** LKMS, plasma protein binding rate, pharmacokinetics, in vitro, in rats

## Abstract

Lekethromycin (LKMS), a novel macrolide lactone, is still unclear regarding its absorption. Thus, we conducted this study to investigate the characteristics of LKMS in rats. We chose the ultrafiltration method to measure the plasma protein binding rate of LKMS. As a result, LKMS was characterized by quick absorption, delayed elimination, and extensive distribution in rats following intramuscular (im) and subcutaneous (sc) administration. Moreover, LKMS has a high protein binding rate (78–91%) in rats at a concentration range of 10–800 ng/mL. LKMS bioavailability was found to be approximately 84–139% and 52–77% after im and sc administration, respectively; however, LKMS was found to have extremely poor bioavailability after oral administration (po) in rats. The pharmacokinetic parameters cannot be considered linearly correlated with the administered dose. Additionally, LKMS and its corresponding metabolites were shown to be metabolically stable in the liver microsomes of rats, dogs, pigs, and humans. Notably, only one phase I metabolite was identified during in vitro study, suggesting most of drug was not converted. Collectively, LKMS had quick absorption but poor absorption after oral administration, extensive tissue distribution, metabolic stability, and slow elimination in rats.

## 1. Introduction

Macrolides are a class of antibiotics which contain a 12–16 carbon-lactone ring in their chemical structure [1], and are widely used against Gram-positive (G^+^) bacteria, and to a lesser extent against Gram-negative (G^-^) bacteria, *Chlamydia*, *Mycoplasma*, and *Legionella*. Due to the emergence and spread of multidrug-resistant bacteria, the development of new drugs for use in animals and humans has become increasingly necessary. In this context, pharmacokinetic studies are an important and indispensable step of drug development [2].

Lekethromycin (LKMS) is a semi-synthetic macrolide whose molecular structure is similar to tulathromycin. Macrolides have high lipophilicity and are broadly dispersed in the blood and in tissues [3], with slightly different pharmacokinetic properties based on their chemical structure. Additionally, the efflux transporter protein P-glycoprotein (P-gp) encoded by the adenosine triphosphate-binding cassette B1 (*ABCB1*) gene limits macrolide absorption in the gut, whereas *ABCB1* mediates macrolide excretion in the bile [4,5]. Tulathromycin has been studied in a wide range of species, including cattle, pigs, goats, and ponies, showing a long terminal half-life; it was demonstrated that tulathromycin concentration peaked at approximately 1 h after subcutaneous administration in cattle and after intramuscular administration in pigs [6,7,8,9] with, respectively, >90% and >80% of bioavailability [10,11], >10 L/Kg of apparent volume of distribution, and 53–68% of plasma protein binding rate [11]. Moreover, tulathromycin was shown to be less metabolized and activate similar metabolic pathways when administered to cattle, pigs, dogs, and rats. Tulathromycin concentration in lung tissue was higher than that in plasma [12], indicating that a single-dose delivery is possible [13]. In addition, the prototype was found among major excreted tulathromycin metabolites (>90%) [14]. Tulathromycin is primarily excreted in the bile and urine [11], with slow excretion rates in cattle and pigs [15]. Tulathromycin is metabolized differently in cattle and pigs (i.e., by *N*-demethylation, *N*-oxidation and the combinations of other metabolic pathways based on oxidation and demethylation) [14]. However, it was virtually completely excreted when in the prototype form, and most of the drug was cleared. The European Medicine Agency reported that 30–50% of tulathromycin in the prototype form was excreted in swine feces, with only approximately 1% excreted in the urine after oral administration (2.5 mg/kg) [14]. 

LKMS, a tulathromycin-derivative and novel macrolide produced independently by our group, had an activity equivalent to that of analogous commercial drugs, showing an MIC_50_ within the range of 0.5–8 μg/mL for common G^+^ bacteria, and 1–8 μg/mL for G^-^ bacteria, respectively. The development of new drugs is urgent to contain the emergence and spread of multidrug-resistant bacteria. The present study aimed to investigate the pharmacokinetic profile of LKMS after po, im, and sc administration in rats. In addition, LKMS metabolism was studied in rat liver microsomes to understand the in vitro pathway. The studies are necessary to more accurately establish its safety and efficacy in rats, if applicable, on its tissue kinetics to understand the absorption and transportation.

## 2. Results

### 2.1. Analytical Method

To determine the rate of LKMS plasma protein binding in rat plasma, an ultrafiltration approach was utilized in this paper. The present investigation evaluated the accuracy and precision of LKMS in the upper layer of filter cup after centrifugation. As shown in Table 1, its recovery ranged from 94.60% to 112.55% for intra-day and from 94.17% to 111.09% for inter-day, with intra- and inter-batch precision values less than 7.74%.

Our lab has developed and validated a reliable and sensitive method to determine LKMS plasma concentration in rats [16], and the method was thoroughly verified for specificity, linearity, accuracy, precision, matrix effect, extraction recovery, dilution integrity, and storage stability, which was still employed to examine the LKMS concentration in this work.

### 2.2. Plasma Protein Binding Rate of LKMS

In this study, the LKMS plasma protein binding rate in rats was measured via ultrafiltration method at concentrations of 10, 200, 800 ng/mL. In order to achieve a suitable ultrafiltration performance for the assessment of plasma binding rate, the effects of incubation time and stability were investigated. The results are summarized in Table 2 and Table 3, suggesting LKMS was stable in rat plasma after 2 h incubation at 37 °C. Meanwhile, at various incubation time intervals, the concentration of LKMS in the upper layer of the filter cup after centrifugation did not demonstrate a significant variation. The rate of LKMS plasma protein binding ranged from 78% to 91% (Table 4). Compared with a concentration of 10 ng/kg, the binding rate of LKMS (91 ± 4%) was significantly lower than the concentration of 800 mg/kg (78 ± 9%).

### 2.3. LKMS Pharmacokinetics Study

**Figure 1:**
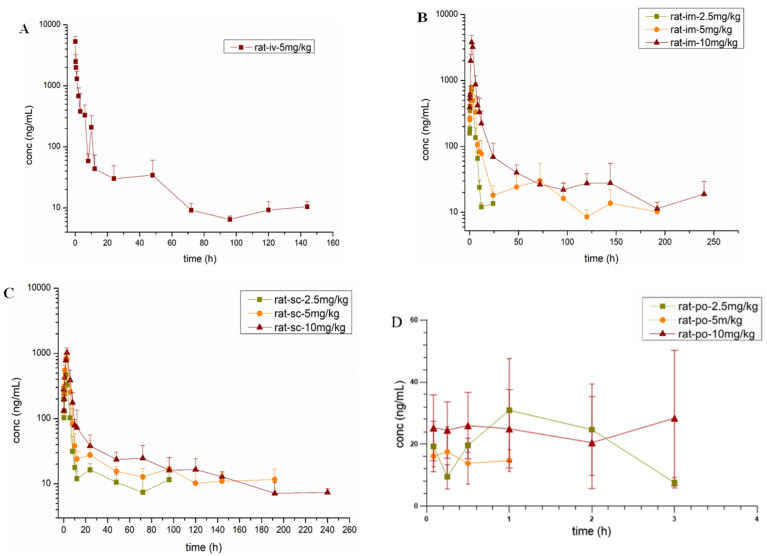
Plasma pharmacokinetic curves of lekethromycin (LKMS) in rats after administration via different routes, regimens, and doses: (**A**) Pharmacokinetic profile in rats after intravenous administration of a single dose at 5 mg/kg; (**B**) Pharmacokinetic profiles in rats after intramuscular administration at 2.5 mg/kg, 5 mg/kg and 10 mg/kg; (**C**) Pharmacokinetic profiles in rats after subcutaneous administration at 2.5 mg/kg, 5 mg/kg and 10 mg/kg; (**D**) Pharmacokinetic profiles in rats after oral administration at 2.5 mg/kg, 5 mg/kg and 10 mg/kg.

The PK parameters and drug-time curves of the LKMS in rat plasma were obtained using Phoenix WinNonlin 8.4 software (Table 5, Figure 1). After 3 h following po administration, LKMS concentrations in plasma were below LOQ, and the data were not meaningful. Meanwhile, the concentrtions in plasma were also below the LOQ after 12 h of im adminitration at a dose of 2.5 mg/kg. However, considering those data and software rules, we estimated po PK parameters by using LOQ instead of the data that cannot be quantified.

The half-life time (T_1/2_) of LKMS after im and sc administration were estimated to range from 48.37 h to 57.54 h and from 64.02 to 136.70 h at three doses of 2.5, 5, and 10 mg/kg, respectively, which indicated that the LKMS elimination rate was low in rats. The time to reach peak plasma concentration (T_max_) was estimated to be 1.8–2.2 h and 2.2–3.0 h after im and sc administration, respectively, which suggested that LKMS was rapidly absorbed in rats. Furthermore, there appeared to be no significant difference between the im and sc routes. The mean residence time (MRT) of LKMS after im and sc administration was 21.46–43.38 h and 28.19–38.07 h, respectively. However, the MRT (43.38 h) at dose of 5 mg/kg was significant different compared with the dose of 2.5 mg/kg (21.46 h) and 10 mg/kg (27.67 h) after im. Additionally, the results also showed significant individual differences. The mean calculated body clearance (Cl) was slow (0.58 ± 0.17 L/kg/h), and the steady state volume of distribution (V_ss_) was 11.60 ± 0.57 L/kg in rats after iv administration. A high absolute im bioavailability was detected in rats with an estimated value ranging from 84% to 139%. The bioavailability ranged from 52% to 74% after sc administration, indicating lower absorption properties compared with im route. The bioavailability revealed the good absorptive capability of LKMS in rats. The po adminitration showed extremly poor absorption, wherein the LKMS concentration was below LOQ within 3 h. Due to the small amout of data in this study, the PK profile following po (2.5 mg/kg) and sc administration were shown by 3 h and 24 h, respectively. The apparent distribution volume values (V_d_) of 32.56–49.60 L/kg for im admnistration and 94.25–99.26 L/kg for sc administration, indicating an apparent distribution in rats via im and sc adminisatrtion. Accordingly, the area under the concentration–time curve (AUC_last_) revealed statically significant changes according to the administration route, with an order of iv > im > sc at dose of 5 mg/kg. Moreover, the confidence interval method was used to evaluate the linear pharmacokinetics in the study, as showed in Table 6. Due to the minor overlap between the confidence interval and judgment interval, those results demonstrated that the AUC_last_ and C_max_ cannot be considered linearly correlated with the administered dose. In short, LKMS was rapidly absorbed, slowly eliminated, widely distributed after sc and im administration, and showed a non-linear pharmacokinetic profile in rats. Notably, the data regarding oral administration was below the LOQ within 3 h. As a result, the bioavailability of po administration was extremely low. Similarly, non-linear pharmacokinetics were observed in po administration.

### 2.4. LKMS In Vitro Metabolism

**Figure 2:**
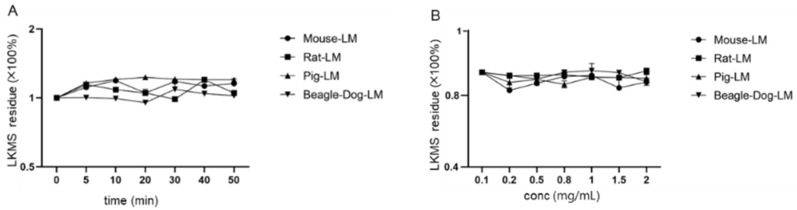

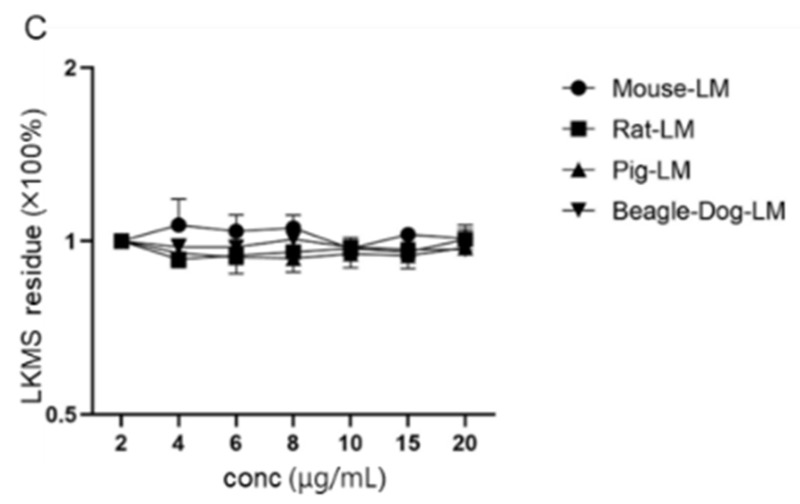
Effect of different incubation periods on the metabolism of lekethromycin (LKMS) in liver microsome (LM) of different species (mouse, rat, pig and beagle dog). (**A**) Incubation time; (**B**) LM protein concentration; (**C**) LKMS concentration.

Subsequently, the metabolic stability of LKMS was assessed in this study. In vitro metabolism of LKMS was not affected by incubation time, liver microsome protein concentration, and substrate concentration. As shown in Figure 2, the half-life of LKMS was greater than 50 min when incubated with different species of liver microsome.

High-resolution mass spectrometry (HRMS) has been widely used for the identification of metabolites in vitro and in vivo [17,18]. Considering the parent drug and its metabolites, full scans and all ion fragmentation acquisitions were conducted using an UHPLC system coupled with an Orbitrap high-resolution mass analyzer to obtain full MS spectra and ddMS2 data [19,20]. The metabolism of LKMS was analyzed using Compound DiscovererTM software with a Fragment Ion Search (fishing) toll in a single workflow.

**Figure 3:**
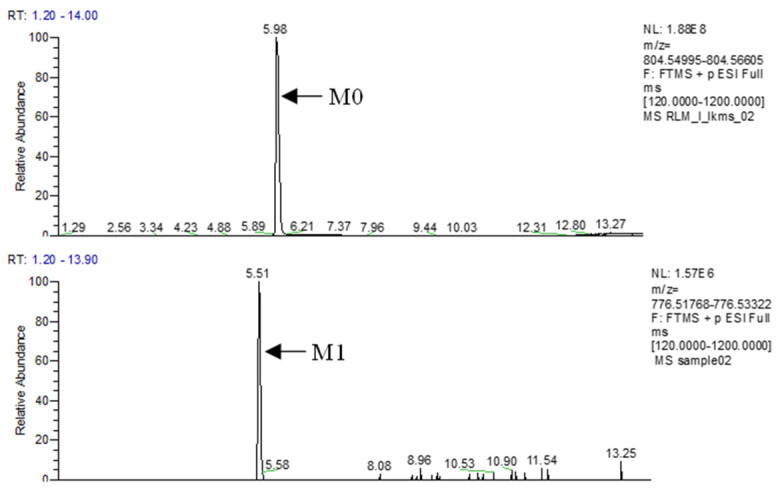
Extracted ion chromatograms of lekethromycin (LKMS) (M0) and its metabolites (M1).

**Figure 4:**
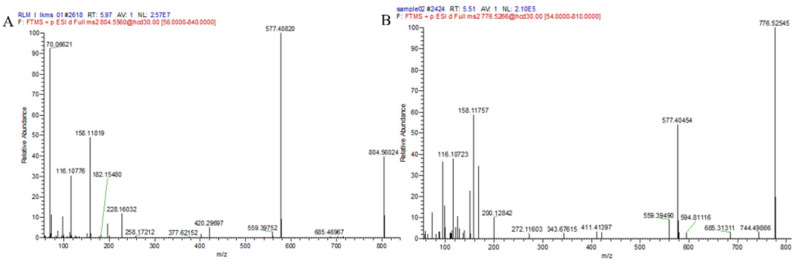
Mass spectra of (**A**) lekethromycin (LKMS) (M0, LKMS prototype) and (**B**) its metabolites (M1, phase I metabolite).

Only one phase I metabolite of M1 was found in liver microsome of different species in this study. No metabolites were associated to phase II biotransformation. The chromatograms of LKMS and its associated metabolites are shown in Figure 3, and mass spectra are shown in Figure 4. The in vitro metabolic pathway of LKMS in the liver microsome system is depicted in Figure 4.

Considering M0, the primary ions (*m*/*z* 804.5602) with the chemical formula C_41_H_77_N_3_O_12_ resulted in the following fragment ions: *m*/*z* 577.4082, *m*/*z* 420.2969, *m*/*z* 228.1603, *m*/*z* 158.1181, and *m*/*z* 116.1077, with a retention time of 5.98 min. Collectively, the chromatograms agreed with the secondary ion mass spectra of the parent drug.

**Figure 5:**
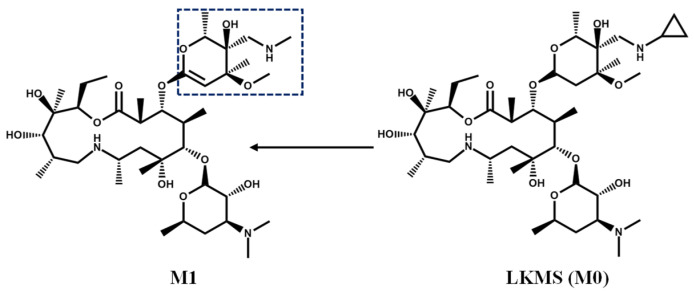
The proposed metabolic pathway of lekethromycin (LKMS) in vitro.

Considering M1, the retention time of primary ions (*m*/*z* 776.52545) was 5.51 min, and the chemical formula was C_39_H_73_N_3_O_12_, with the following fragment ions: *m*/*z* 577.4045, *m*/*z* 158.1175, *m*/*z* 116.1072, and *m*/*z* 200.1284. Inferred from fragment ions *m*/*z* 577.4045, 158.1175, and 116.1072, it could be inferred that the lactone ring and the aminoglycan fraction did not change. However, *m*/*z* 200.1284, with a 28-Da difference compared to the fragment ion *m*/*z* 228.1603, was found. Thus, as shown in Figure 5, M1 was considered the clathrate structure of LKMS which underwent decyclopropylation, *N*-methylation, and hydroxylation of the oxygen-containing five-carbon lactone ring.

## 3. Discussion

To the best of our knowledge, this study is the first to describe the pharmacokinetics of LKMS in rats. The present investigation was carried out to obtain the pharmacokinetics of LKMS when administered po, im, and sc in rats in light of the earlier work in our lab that this medicine is safe and efficient.

Plasma protein binding is of vital importance in the pharmacokinetics and pharmacodynamic of drugs [17]. Many methods in vitro have been developed for assessing drug-protein binding, including equilibrium dialysis, ultrafiltration, and ultracentrifugation [18]. The plasma protein binding rate was determined via an ultrafiltration method rate since it is less time consuming and easy to handle despite its non-specific binding problem [19,20], and it was found to be ranged 78–91%. A high plasma protein binding rate typically indicates that there is less medicine in free state in the plasma and that a drug–drug interaction should be taken into account when co-administrating [21]. Unbound drugs in plasma can easily reach the target organ compared with the bunded fraction. Similar to other macrolides, the plasma protein binding rate in rat plasma decreased with increasing LKMS concentration, indicating that LKMS would mostly bind to α-acidic glycoprotein rather than albumin. However, there is a shortage in the present study since we examined the LKMS concentration in concentrated phase rather than ultrafiltrate.

In the current study, the dose of LKMS varied by extravascular routes. Additionally, a study on iv pharmacokinetics was conducted previously to determine the parameters, including V_ss_, Cl, and bioavailability. The V_ss_ of LKMS was 11.60 ± 0.57 L/kg, and it is possible that the lower volume of distribution was due to the relatively high plasma protein binding rate in rats, reaching C_max_ at 1.80–2.20 h and 2.00–3.00 h, respectively. T_1/2_ of LKMS ranged from 2d to 6d in this study, which is comparable to the pharmacokinetic character of tulathromycin (T_1/2_ of 4–6 d) [7,8,9]. A long LKMS residence time in plasma (>17 h) was observed, indicating an extended action period. Furthermore, LKMS displays a significant difference in mean values of bioavailability. When given via the im and sc routes, respectively, the bioavailability of LKMS was 52–77% and 84–139%, which was comparable to that of tulathromycin. However, when LKMS was administered orally, a significantly lower bioavailability was observed. When given as a single gavage, tulathromycin was found to have an equally poor bioavailability [15]. However, it is also important to consider the differences in chemical structure between LKMS and tulathromycin. In addition, it was found that LKMS plasma concentrations were below LOQ after 3 h po administration in rats. As a result, the po bioavailability of LKMS was almost negligible. This phenomenon previously appeared in a pharmacokinetic study of tulathromycin [22]. Due to its poor permeability and the fact that it is the substrate of efflux pumps P-gp and MRP2, which have been examined in the Caco-2 cell model, there may be a feasible explanation for the limited oral bioavailability of LKMS (unpublished data). Im and sc administration can prevent medication metabolism in the digestive system when compared to po administration. Given that LKMS is unstable in solutions with a pH lower than 2, its stability in the digestive system must be taken into account. It is unlikely that po administration was chosen in contrast to the im and sc administration. Moreover, this study demonstrated that the AUC_last_ and C_max_ cannot be considered as linearly correlated with the administered dose since there is a minimal overlap between the confidence interval and judgment interval.

The results of the metabolite studies suggested most of the parent LKMS was not biotransformed in rats. It is assumed that LKMS is mainly eliminated unchanged. Similarly, Pfizer reported that tulthromycin is not converted and is excreted by biliary and renal excretion [11]. Phase I metabolism is the conversion of lipophilic compounds into highly polar derivatives that are rapidly excreted through a specific metabolic enzyme system. As demonstrated by our results, LKMS has a moderately lipophilic LogD_7_._4_ of 2.27. It is not easily metabolized and may result in low binding to metabolic enzymes [23]. It is noteworthy that no metabolite connected to phase II biotransformation was found during in vitro study. Similar to tulathromycin, tulathromycin was metabolized at a lower level and was primarily eliminated in pigs [14]. Low clearance of LKMS in rats is consistent with LKMS being insignificantly degraded and behaving with high metabolic stability [16]. It was therefore hypothesized that LKMS does not interact with metabolic enzymes and undergoes extensive metabolism.

In conclusion, LKMS was demonstrated to have a high protein binding rate (78–91%), and pharmacokinetic results indicated that LKMS had fast absorption, slow elimination, and wide distribution after im, sc, and po administration in rats. The bioavailability of im and sc was higher than 84% and 52%, respectively, whereas the absorption of po administration is insufficient. In vitro, LKMS and its metabolites had high metabolic stability in different species of liver microsomes. Additionally, this investigation only identified one phase I metabolite, suggesting most of the parent LKMS was not biotransformed in rats. Further studies are necessary to determine LKMS safety and efficacy, as well as tissue kinetics to fully understand its absorption and transport in rats.

## 4. Materials and Methods

### 4.1. Chemicals and Reagents

LKMS standard (batch no. D20170101, purity ≥ 97%) was obtained from Henan Pulike Biological Engineering Co., Ltd. (Luoyang, Henan, China). Gamithromycin was purchased from Sigma–Aldrich (St. Louis, MO, USA). LC-MS grade methanol (MeOH), acetonitrile (ACN) and formic acid (FA) were supplied by Fisher Scientific (Pittsburgh, PA, USA). Ultrapure water was obtained from a Millipore Milli-Q purification system (Bedford, MA, USA). NADPH and UDPGA were purchased from Sigma–Aldrich. Liver microsomes were purchased from RILD Co., Ltd. (Shanghai, China). PBS tablets were purchased from Fisher Scientific (Pittsburgh, PA, USA). Amicon^®^ Ultra 0.5 mL Centrifugal Filters were purchased from Sigma–Aldrich.

### 4.2. Determination of LKMS Plasma Concentration

Briefly, 10 μL of LKMS standard and 10 μL of internal standard were transferred to 180 μL of plasma, followed by vortexing for 10 s. Subsequently, 600 μL of acetonitrile was added to precipitated protein, followed by centrifugation at 14,000 rpm for 20 min at 4 °C. The supernatant was subsequently evaporated at room temperature using nitrogen purge. The sample was reconstituted using 400 μL of injection solution (ACN: 1% FA∙H_2_O = 1:9) and transferred to injection vials for UPLC-MS/MS detection.

### 4.3. Determination of LKMS Plasma Protein Binding Rate

An ultrafiltration method was chosen to determine the plasma protein binding rate of LKMS using Amicon^®^ Ultra-0.5 Centrifugal Filters (10 kDa cutoff). To obtain plasma containing LKMS at different final concentrations (10, 200, and 800 ng/mL), 2 μL of LKMS work solution (with acetonitrile at approximately 1% of the total volume) was added to 200 μL of original rat plasma. The plasma was incubated at 37 °C for 30 min to reach an equilibrium of drug prior to use in the plasma protein binding assay. Then, stability of LKMS throughout different incubation periods was assessed.

Ultrafiltration tubes were pre-wet in PBS buffer (pH 7.4) for 5 min, then agitated to dry, and the concentrate collection cup was weighed to determine the tube’s initial weight (m1). Then, 200 μL of LKMS-containing plasma was placed in ultrafiltration tubes for 20 min, followed by centrifugation for 5 min at 12,000 rpm. The concentrate inner tube was inserted in reverse into the pre-weighed concentrate collection tube, centrifuged for 2 min at 12,000 rpm, and the concentrate collection tube was weighed a second time (m2). Protein leakage was determined using 10% perchloric acid solution, and total plasma concentration (C1) and concentrate concentration (C2) were calculated. The plasma protein binding rate was estimated according to the formula below:

PPB = [C2 × (m2 − m1)]/(0.2 × C1)(1)

### 4.4. Animal Study Design

In total, 60 Sprague Dawley rats (male/female) were submitted to fasting for 12 h prior to the start of the experiment and were given free access to water. Rats were weighed prior to LKMS administration to determine the dose. Based on the study design shown in Table 7, rats were given the exact doses of LKMS. Blood samples (0.3–0.4 mL) were collected at 0 h (pre-administration phase) and 5 (min), 0.25, 0.5, 1, 2, 3, 6, 8, 10, 12, 24, 48, 72, 96, 120, 144, 192, 240 h after administration. Animal experiments were approved by the Review Committee of Animal Care and Use of China Agricultural University (14408-19-R-026). Blood samples were centrifuged at 4000 rpm for 10 min and stored in a freezer at −20 °C until further analysis.

### 4.5. In Vitro Metabolism

The total incubation system (500 μL) consisted of PBS buffer (100 mM, pH 7.4), liver microsomes (2 mg/mL), LKMS (10 μg/mL) and NADPH or UDPGA (2 mM). After pre-heating for 5 min at 37 °C, the reaction started by the addition of freshly produced NADPH or UDPGA. After incubation for 1 h at 37 °C, the reaction was terminated by the addition of an equal volume of ice-cold acetonitrile containing an internal standard, followed by vortexing and shaking for 1 min, and then by centrifugation at 14,000 rpm for 20 min. Then, the supernatant was transferred to injection vials for further analysis.

Tandem mass spectrometry was performed with a Q-Exactive Orbitrap MS (Thermo Fisher, Waltham, MA, USA) in the positive ion mode via fullmass-ddms2, employing a heated electrospray ionization source for the ionization of target compounds. MS/MS parameters employed were the following: spray voltage, 3.50 kV; sheath gas flow rate, 40 mL/min; auxiliary gas flow rate, 10 mL/min; capillary temperature, 320 °C; auxiliary gas heater temperature, 350 °C; scan modes, full mass-ddms2 with NCE (15, 30, and 45 eV). Xcalibur 4.1 software (Thermo Fisher Scientific, Waltham, MA, USA) was used to collect MS/MS data. Compound DiscoverTM software was used to assess raw MS data files.

### 4.6. Statistical Analysis Method

The lekethromycin plasma concentration versus time data for each rat was subject to noncompartmental analysis using Phoenix WinNonlin 8.4 software. The difference between groups were compared using SPSS software. Values were considered significantly different if the *p*-value was <0.05.

The confidence interval method was used to assess linear pharmacokinetics [24,25,26]. The regression equation can be expressed as follows:

(2) $$\ln\left(y\right)=\alpha +\beta \times \ln\left(Dose\right)$$

The following equation can be used to calculate the judgement interval:

(3) $$1+\frac{\ln\left(\theta_{L}\right)}{\ln\left(r\right)}\;<\;\beta \;<\;1+\frac{\ln\left(\theta_{H}\right)}{\ln\left(r\right)}$$

The equation for confidence interval calculated can be expressed as follows:

(4) $$S_{y\cdot x}=\sqrt{\frac{\sum \left(y-\bar{y}\right)\sum {\left(y-\;\bar{y}\right)}^{2}}{n-2}}$$

(5) $$\;S_{b}=\frac{S_{y\cdot x}}{\sum {\left(x-\bar{x}\right)}^{2}}$$

b − t_α(v)_*S_b_* ≤ β ≤ b − t_α(v)_*S_b_* (6)

The codes used in the text have the following meanings: *H* is the highest administrated dose and *L* is the lowest administrated dose, hence *r* = *H*/*L*. *θ_H_*: upper confidence interval; *θ_L_*: lower confidence interval. The pharmacokinetic parameters can be considered as linearly correlated with the administered dose when the (1 − α) percentage confidence interval of the slope β falls exactly in the judgment interval.

## Figures and Tables

**Table 1:** Intra-day (*n* = 16) and inter-day (*n* = 18) precision and accuracy of lekethromycin (LKMS) in solution at various concentrations (mean ± standard deviation).

LKMS Concentration (ng/mL)	Intra-Day (*n* = 6)		Inter-Day (*n* = 18)	
	Accuracy (%)	Precision (%)	Accuracy (%)	Precision (%)
10	94.60 ± 1.02	7.47	94.17 ± 1.06	5.46
200	112.55 ± 1.05	4.75	111.09 ± 2.06	3.29
800	95.52 ± 1.01	4.32	93.93 ± 1.05	3.36

**Table 2:** Lekethromycin (LKMS) concentration at different incubation periods at 37 °C in rat plasma (*n* = 5).

LKMS Solution Concentration (ng/mL)	LKMS Concentration (ng/mL) (*n* = 5)				Relative Standard Deviation (%)
	0 h	0.5 h	1 h	2 h	
10	8.44 ± 0.04	8.76 ± 0.05	9.44 ± 0.3	9.66 ± 0.5	6.02
200	196.21 ± 9.15	195.81 ± 8.21	197.69 ± 7.64	198.59 ± 16.39	1.57
800	714.92 ± 40.17	800.23 ± 45.24	802.56 ± 50.17	805.61 ± 62.17	6.78

**Table 3:** Lekethromycin (LKMS) concentration in concentrated solution at different incubation periods (*n* = 5).

LKMS Solution Concentration (ng/mL)	LKMS Concentration (ng/mL) (*n* = 5)			Relative Standard Deviation (%)
	0.5 h	1 h	2 h	
10	17.70 ± 0.78	17.70 ± 0.61	15.75 ± 1.30	7.90
200	373.51 ± 44.70	372.59 ± 9.45	357.75 ± 10.64	7.80
800	871.00 ± 66.13	913.00 ± 23.04	941.11 ± 40.59	6.99

**Table 4:** Plasma protein binding rate for LKMS in rats.

LKMS Concentration (ng/mL)	Protein Binding Rates (%)
10	91 ± 4
200	87 ± 6
800	78 ± 9

**Table 5:** Pharmacokinetic parameters of lekethromycin (LKMS) after intravenous (5 mg/kg), intramuscular (2.5, 5, and 10 mg/kg) and subcutaneous (2.5, 5, and 10 mg/kg) administration in rats.

PK Parameters	iv (5 mg/kg)	im			sc		
		2.5 mg/kg	5 mg/kg	10 mg/kg	2.5 mg/kg	5 mg/kg	10 mg/kg
T_1/2λZ_ (h)	32.33 ± 14.63	48.37 ± 2.76b	57.54 ± 10.07a	56.76 ± 11.83a	136.70 ± 15.23b	131.93 ± 14.44b	64.02 ± 12.68a
T_max_	-	1.80 ± 0.45a	2.00 ± 0.00a	2.20 ± 0.45a	2.20 ± 0.45b	2.00 ± 0.55b	3.00 ± 0.00a
C_max_ (μg/mL)	5.73 ± 1.39	0.74 ± 0.19b	0.83 ± 0.11b	4.30 ± 1.11a	0.48 ± 0.04b	0.91 ± 0.13a	1.03 ± 0.18a
AUC_last_ (h·μg/mL)	8.91 ± 2.31	3.99 ± 0.17c	7.53 ± 1.56b	24.89 ± 5.00a	3.47 ± 0.27c	6.61 ± 1.11b	9.33 ± 1.48a
AUC_INF_obs_ (h·μg/mL)	9.13 ± 2.37	4.74 ± 1.17b	8.44 ± 1.64b	26.81 ± 5.43a	4.52 ± 0.29c	7.93 ± 1.25b	9.99 ± 1.61a
V_z_obs_ (L/kg)	25.56 ± 7.93	-	-	-	-	-	-
Vz_F__obs_ (L/kg)	-	32.56 ± 14.37b	49.60 ± 7.37a	43.82 ± 18.81a	94.25 ± 16.89a	83.92 ± 7.31b	99.26 ± 12.07a
V_ss_ (L/kg)	11.60 ± 0.57	-	-	-	-	-	-
Cl__obs_ (L/h/kg)	0.58 ± 0.17	-	-	-	-	-	-
Cl_F__obs_ (L/h/kg)	-	0.55 ± 0.13a	0.61 ± 0.11a	0.39 ± 0.09b	0.52 ± 0.08b	0.64 ± 0.09b	1.11 ± 0.25a
MRT_last_ (h)	17.38 ± 7.71	21.46 ± 9.40b	43.38 ± 10.50a	27.67 ± 7.77b	28.19 ± 1.89a	31.09 ± 8.20a	38.07 ± 3.52a
F (%)	-	89	84	139	77	74	52

T_1/2λz_, elimination half-life; T_max_, time to reach peak plasma concentration; C_max_, plasma peak concentration; AUC_last_, area under the concentration–time curve from 0 to the last time point; AUC_INF_obs_, area under the concentration–time curve from 0 to infinity. V_Z_obs_, apparent volume of distribution (for intravascular); V_Z_F_obs_, apparent volume of distribution (for extravascular); V_ss_, steady state volume of distribution (for intravascular); Cl__obs_, apparent body clearance (for intravascular); Cl__F_obs_, apparent body clearance (for extravascular); MRT_last_, mean residence time. F, absolute bioavailability. Values were considered significantly different if the *p*-value was <0.05. The different symbols such as “a, b, c” mean significantly different statistically between different administration does.

**Table 6:** Confidence interval criteria for the evaluation of linear pharmacokinetics for lekethromycin (LKMS) after intramuscular and subcutaneous administration.

Linear Pharmacokinetics Criteria	Intramuscular Administration		Subcutaneous Administrations	
	C_max_	AUC_last_	C_max_	AUC_last_
Linear regression formula	y = 1.303x + 6.9704	y = 1.2833x + 5.646	y = 0.6031x + 7.7227	y = 7.46x + 0.72
Confidence interval	[0.81, 1.80]	[0.69, 1.68]	[0.11, 1.10]	[0.05, 1.04]
Judgement interval	[0.84, 1.16]	[0.74, 1.26]	[0.84, 1.16]	[0.74, 1.26]

C_max_, plasma peak concentration, μg/mL; AUC_last_, area under the concentration–time curve from zero to the last time point, h·μg/mL.

**Table 7:** Pharmacokinetic dose regimen of lekethromycin (LKMS) in rats.

Group	Animal Number Code	Administration Route	Dose of LKMS (mg/kg)
1	1–6	Intravenous	5
2	7–12		2.5
3	13–18	Intramuscular	5
4	19–24		10
5	25–30		2.5
6	31–36	Subcutaneous	5
7	37–42		10
8	43–48		2.5
9	49–54	Oral	5
10	55–60		10
